# COVID-19 y enfermedad pulmonar pediátrica: Experiencia en un centro de atención terciaria en Sudáfrica

**DOI:** 10.1159/000515615

**Published:** 2021-03-09

**Authors:** Diane M. Gray, Mary-Ann Davies, Leah Githinji, Michael Levin, Muntanga Mapani, Zandiswa Nowalaza, Norbertta Washaya, Aamir Yassin, Marco Zampoli, Heather J. Zar, Aneesa Vanker

**Affiliations:** 1 ^a^Departamento de Pediatría y Salud Infantil, Universidad de Ciudad del Cabo, Ciudad del Cabo, Sudáfrica; 2 ^b^Consejo de Investigación Médica (MRC), Unidad de Salud Infantil y Adolescente, Universidad de Ciudad del Cabo, Ciudad del Cabo, Sudáfrica; 3 ^c^Escuela de Salud Pública y Medicina Familiar, Universidad de Ciudad del Cabo, Ciudad del Cabo, Sudáfrica

**Keywords:** COVID-19, SARS-CoV-2, Pediatría, Enfermedad pulmonar, Enfermedad pulmonar crónica en la infancia, Países con ingresos bajos o medios, Tuberculosis pediátrica

## Abstract

La pandemia de COVID-19 resultó en una rápida diseminación global, con profundos impactos en los sistemas de salud. Aunque los datos pediátricos muestran de manera consistente un cuadro clínico más leve, se ha identificado que la enfermedad pulmonar crónica es un factor de riesgo para la hospitalización y para desarrollar una enfermedad grave. En África, continente formado predominantemente por países con ingresos bajos o medios (LMIC), la elevada prevalencia de VIH, tuberculosis, desnutrición y hacinamiento aumenta aún más los riesgos a la salud. En este trabajo se revisa la literatura sobre COVID-19 y enfermedad pulmonar crónica en niños, y relata nuestra experiencia en un centro de atención pulmonar pediátrico en Ciudad del Cabo, Sudáfrica. Los datos epidemiológicos en Sudáfrica confirman una baja prevalencia de la enfermedad grave, donde los pacientes < 18 años comprenden 8% de todos los casos diagnosticados de COVID-19 y 3% de todas las admisiones por esa causa. Se encontró una reducción en la admisión hospitalaria por otras infecciones del tracto respiratorio inferior. Aunque el servicio de pulmonología atiende niños con una amplia variedad de condiciones respiratorias crónicas, incluyendo bronquiectasias, fibrosis quística, asma, enfermedad pulmonar intersticial y pacientes con traqueostomías, no se observó un incremento significativo en las admisiones por COVID-19, y en quienes desarrollaron COVID-19, el curso de la enfermedad no fue grave. La evidencia actual sugiere que la preexistencia de una enfermedad respiratoria en niños no parece ser un factor de riesgo significativo para el COVID-19 grave. Aún se requieren datos longitudinales para evaluar el riesgo en niños con inmunosupresión y enfermedades pulmonares intersticiales. Los impactos indirectos de la respuesta a la pandemia en la salud respiratoria de los niños son notables, y es muy probable que aún deban comprenderse y cuantificarse. Garantizar el acceso de los niños a servicios preventivos y de cuidado completos durante este tiempo es prioritario.

## Antecedentes

La pandemia de COVID-19, causada por un nuevo coronavirus (SARS-CoV-2), surgió en Wuhan, China, a finales de 2019 y rápidamente se distribuyó por todo el globo, sin excluir ningún continente; el primer caso en África se reportó en Egipto en febrero de 2020. Para el 29 de septiembre de 2020, se habían reportado 33,249,563 casos en el mundo, con 1,000,040 muertes y 216 países afectados. En África se habían reportado 1,179,152 casos y 25,760 muertes, 3.5 y 2.6% del total global, respectivamente [1]. Conforme la pandemia se extendió, con alta mortalidad, predominantemente en pacientes respiratorios, representando una carga significativa para los sistemas de salud, la Organización Mundial de la Salud (OMS) externó varias preocupaciones para la región de África, donde la prevalencia de VIH y otras enfermedades infecciosas como la tuberculosis (TB) aún es alta, y donde varios países aún dependen de sistemas de salud precarios [2]. En el continente africano, el mayor número de casos de coronavirus confirmados a la fecha (septiembre de 2020) se ha reportado en Sudáfrica [1]. Los datos en la población pediátrica en todo el mundo han mostrado de manera consistente una tasa de infección más baja y un cuadro clínico más leve en niños. En una revisión de más de 70,000 casos chinos de COVID-19, <1% eran niños menores de 10 años, con síntomas distintos y menos graves que en los adultos [3]. De manera similar, un análisis muy extenso de cerca de 150,000 casos de COVID-19 en los Estados Unidos de América encontró que sólo 1.7% fueron <18 años; de ellos, un número muy pequeño requirió ingresar a la UCI y se registró una mortalidad extremadamente baja [4]. Sin embargo, datos de más de 400 niños obtenidos en varios centros hospitalarios en Latinoamérica mostraron una forma más grave de COVID-19; 12.7% de los niños incluidos requirieron ingresar a la UCI, y se diagnosticó una alta proporción de síndrome inflamatorio multisistémico pediátrico (MIS-C), indicando una forma más grave de la enfermedad en niños latinos/hispanos o en niños provenientes de niveles socioeconómicos más bajos [5]. Otros países con ingresos bajos y medios (low and middle income countries, LMIC) también han reportado un número menor de casos de COVID-19 y una menor gravedad en niños que en adultos [6]. La enfermedad pulmonar crónica es una de las condiciones médicas preexistentes más comunes que se han reportado en casos pediátricos de COVID-19 en hospitales, tanto en Europa como en Sudáfrica [7, 8]. Se le ha identificado como factor de riesgo para la forma grave de COVID-19 y la muerte en adultos [9, 10]. En niños y adolescentes, la enfermedad respiratoria crónica se ha asociado con el ingreso a la UCI, y se ha asociado con un riesgo aumentado, con un OR de 3.1 (IC: 1.2–8.2), para el ingreso a UCI de pacientes pediátricos [7]. Similarmente, en Sudáfrica, el asma y la enfermedad respiratoria crónica se reportan con frecuencia como factores subyacentes de riesgo en pacientes pediátricos de COVID-19 hospitalizados [8]. Esto ha causado mayor ansiedad en niños con enfermedad pulmonar crónica y en sus padres [11], lo que resalta la importancia de entender mejor el riesgo que sufren estos niños. El impacto de una enfermedad pulmonar preexistente en la gravedad del COVID-19 ha sido una preocupación particular en LMICs, donde hay una gran carga de factores de riesgo adicionales para padecer enfermedad respiratoria grave, como VIH, TB, desnutrición y hacinamiento [12, 13].

Además, los efectos indirectos del COVID-19 en la salud de los niños no son irrelevantes [6, 14]. El intento de contener la propagación de la infección por SARS-CoV2 ha llevado a muchos países, Sudáfrica entre ellos, a implantar políticas de «confinamiento», que tienen grandes repercusiones socioeconómicas, incluyendo restricciones en el acceso al cuidado rutinario de la salud [2, 15, 16, 17]. El desvío de los recursos destinados al cuidado de la salud hacia la atención del COVID-19, incluyendo la reducción de servicios clínicos regulares para enfermedades crónicas y el acceso a las instalaciones de atención primaria, ha significado que los niños tienen acceso limitado a la atención rutinaria e incluso a los programas de inmunización [16, 17, 18]. El temor de contraer COVID-19 en centros de atención médica ha dificultado aún más la asistencia sanitaria. Se ha estimado que el impacto de la reducción de intervenciones esenciales en salud materno-infantil derivada del COVID-19 en LMICs contribuirá a un incremento de 9.8–44.7% en el número de muertes de niños menores de 5 años por mes, y un incremento de 8.3–38.6% en la mortalidad materna por mes [14].

El objetivo de este trabajo es revisar la literatura sobre COVID-19 en niños con enfermedad pulmonar crónica en el mundo, y presentar nuestra experiencia en un centro pulmonar pediátrico en Ciudad del Cabo, provincia de Western Cape, Sudáfrica.

## Epidemiología del COVID-19 en Sudáfrica

Aunque no hay una base de datos pública global con información desagregada por edad y solamente 42 países (aproximadamente 1 de cada 4) publican datos sobre COVID-19 desagregados por edad, se reconoce ampliamente que la prevalencia de COVID-19 y de casos graves de la enfermedad en pacientes <20 años es menor que en adultos, si bien varía considerablemente entre países, desde 1% del total de casos en España a 23% en Paraguay [19]. Hay datos extremadamente limitados y más granulares sobre COVID-19, desagregados por edad, al nivel de país. Hasta la fecha, los estudios de vigilancia mediante pruebas de anticuerpos de todas las infecciones por SARS-CoV-2 por grupo de edad han sido relativamente limitados, y algunos sugieren menores tasas de infección en niños, mientras que otros no encontraron diferencias (20–22). En Sudáfrica, hasta el 5 de septiembre de 2020, los pacientes <20 años representaban ∼8% de 638,517 casos positivos confirmados de COVID-19, y los pacientes de 15–19 años constituían casi la mitad de éstos. El riesgo acumulativo de incidencia de casos de COVID-19 confirmados en laboratorio por 100,000 habitantes se incrementó con la edad en los niños, de 130 (edad <5 años) a 520 (15–19 años), y fue considerablemente más baja que la incidencia total acumulativa para el país, de 1086 casos/100,000 [8].

Los datos de la provincia de Western Cape en Sudáfrica confirman una incidencia muy baja de COVID-19 grave en niños. Hasta el 25 de septiembre de 2020 había 6336 casos positivos de COVID-19 en pacientes <20 años, que constituían 6% de todos los casos diagnosticados en la provincia, 658 ingresos hospitalarios, 27 ingresos a la UCI y 24 muertes (0.6% de todas las muertes por COVID-19 en la provincia), lo que da una tasa de fatalidad en casos diagnosticados de 0.4%. La evolución en el tiempo del número de casos siguió tendencias similares a la de los adultos, con un pico de casos nuevos diagnosticados a finales de junio de 2020 y un descenso gradual subsecuente. El síndrome inflamatorio multisistémico pediátrico (MIS-C) se convirtió hace poco en una enfermedad reportable en Sudáfrica. Se publicó una serie de 23 casos de MIS-C en los dos hospitales de nivel terciario que ofrecen atención pediátrica, sin que se hayan registrado muertes [23].

Varios estudios de modelado han sugerido que, aunque el COVID-19 por sí mismo es esencialmente una enfermedad leve en niños, el efecto indirecto del COVID-19 debido a las interrupciones en los servicios puede tener impactos sustanciales en la salud materno-infantil, con posibles aumentos en la incidencia de varias enfermedades y en la mortalidad infantil. La cobertura nacional de inmunización cayó durante el confinamiento en Sudáfrica de 82% en abril de 2019 a 61% en abril de 2020, con descensos particularmente preocupantes en la cobertura contra el sarampión, de 77% a 55%, especialmente en Western Cape, el lugar donde inicialmente el número de casos de COVID-19 creció con mayor rapidez, y donde la cobertura de la vacuna contra el sarampión sólo alcanzaba 48% en abril de 2020 [18]. También se ha expresado preocupación por la menor cobertura de anticonceptivos, de interrupciones seguras del embarazo, del diagnóstico de VIH y de la cobertura de ART en el embarazo, con un posible incremento en la transmisión materno-infantil, así como en la desnutrición debido a las graves consecuencias económicas del confinamiento [15].

## Planeación y preparación para la pandemia e implicaciones en la atención respiratoria

El Hospital Infantil Memorial de Guerra de la Cruz Roja (RCWMCH) en Ciudad del Cabo es un hospital dedicado, de nivel terciario y de referencia, que ofrece una amplia variedad de servicios de especialidad a pacientes pediátricos y está afiliado a la Universidad de Ciudad del Cabo, Sudáfrica [24]. La unidad de neumología pediátrica atiende a niños con una gran variedad de condiciones respiratorias, tanto agudas como crónicas, mediante servicios intra- y extrahospitalarios. La pandemia de COVID-19 ha requerido adaptar los servicios para continuar prestando atención a niños con enfermedades respiratorias durante este tiempo [25].

En Sudáfrica, la aplicación temprana de un confinamiento «fuerte» se acompañó con la adopción temprana y generalizada de medidas de salud pública, incluyendo el distanciamiento social, la desinfección estricta de las manos y el uso de mascarilla. El transporte público regular se detuvo y los servicios de salud no-urgentes se limitaron para destinar recursos a los centros de detección y tratamiento de COVID-19, mientras que las oficinas que prestan servicios sociales cerraron; a esta respuesta se ha atribuido en parte el menor número de casos y las bajas tasas de mortalidad en comparación con Europa y EE. UU. En el hospital pediátrico, las medidas incluyeron la cancelación de visitas no-esenciales, servicios de radiología limitados, el cese de las pruebas de función pulmonar, el cierre de camas pediátricas, la transferencia de personal al servicio de adultos y de COVID-19 y el diseño de rutas grupales dentro del hospital que redujeran el riesgo de transmisión de COVID-19, a expensas de la continuidad en el cuidado. Todos estos factores podrían afectar el diagnóstico oportuno, la atención y el seguimiento de enfermedades respiratorias.

## COVID-19: impacto en infecciones agudas del tracto respiratorio inferior en niños

En varios países se ha descrito una caída notable en el ingreso de pacientes pediátricos por infecciones agudas del tracto respiratorio inferior (acute lower respiratory tract infections, LRTI) distintas de COVID-19, particularmente de LRTI viral [26, 27, 28]. En el hemisferio sur, la pandemia de COVID-19 coincidió con la temporada anual de infecciones respiratorias virales de 2020. Los datos sobre el ingreso de pacientes pediátricos a la UCI (PUCI) en un extenso registro en Latinoamérica mostró 83% menos ingresos PUCI por LRTI en 2020 con respecto al promedio de 2018/2019 en el mismo periodo, especialmente para el virus sincitial respiratorio (VSR) y la influenza [29]. De manera similar, en Sudáfrica se ha visto un marcado descenso en las tasas de incidencia y hospitalización por LRTI durante el pico usual de ingresos en el invierno (Olsen et al., en revisión). La LRTI por VSR en particular se ha visto marcadamente reducida en comparación con los cinco años anteriores. Igualmente, se han observado grandes reducciones en la incidencia de influenza y en las hospitalizaciones por esa causa, y no se han registrado ingresos hospitalarios de pacientes pediátricos por influenza desde mayo de 2020. Esta marcada reducción en el número esperado de casos de LRTI también se ha observado en China y Hong Kong, y la vertiginosa caída en la incidencia de infecciones asociadas con los virus coincidió con el inicio de las medidas de salud pública relacionadas con el COVID-19 [26].

## COVID-19 en niños con enfermedad pulmonar preexistente y comorbilidades (Tabla 1)

### Bronquiectasias y bronquiolitis obstructiva

Los individuos con enfermedad pulmonar crónica preexistente, incluyendo la enfermedad pulmonar obstructiva crónica (EPOC), tienen mayor riesgo de sufrir COVID-19 grave si son adultos [10, 41]. La bronquiolitis obstructiva (BO) es una enfermedad obstructiva crónica de las vías respiratorias caracterizada por una obstrucción del flujo de aire en las vías respiratorias menores. Una lesión en el epitelio de las vías respiratorias conduce a cambios inflamatorios neutrofílicos, cicatrización y a una posible progresión a una enfermedad pulmonar obstructiva crónica [42]. Los pacientes con BO están en riesgo de exacerbaciones graves, con infecciones respiratorias intercurrentes, de ahí la preocupación por el riesgo aumentado de padecer la forma grave de COVID-19 [43]. Además, los pacientes con enfermedades pulmonares crónicas muestran un aumento en la expresión epitelial de los receptores a la ACE2, a los cuales se une el SARS-CoV-2 para ingresar a las células [44]; la mayor morbilidad y mortalidad en la enfermedad pulmonar crónica puede agravarse por la preexistencia de una reserva pulmonar reducida [10]. Varios estudios de cohorte pediátricos han descrito que la obstrucción crónica de las vías respiratorias es una comorbilidad asociada con la gravedad del COVID-19 [30, 31]. Se ha reportado la presencia subyacente de enfermedad obstructiva crónica de las vías respiratorias, más comúnmente asma y displasia broncopulmonar, en 5% de los pacientes <18 años en Europa ingresados con COVID-19, y en 13% de los niños que requirieron ingresar a la UCI [7]. Las bronquiectasias fueron poco comunes, lo que refleja la baja prevalencia de estas condiciones en países con ingresos altos.

El servicio de consulta externa para pacientes respiratorios en el RCWMCH da seguimiento a una cohorte de aproximadamente 120 niños con bronquiectasias o BO. Desde que se reportó el primer caso de COVID-19 y se iniciaron las medidas de confinamiento en Sudáfrica, en marzo de 2020, se dio preferencia al seguimiento telefónico de los pacientes. Durante este tiempo, la división de neumología no experimentó aumento alguno en el ingreso de pacientes con bronquiectasia, ni se incrementaron las exacerbaciones; ninguno de estos pacientes requirió admisión por COVID-19. Actualmente se están recabando datos sobre el ingreso de pacientes pediátricos en múltiples centros en Sudáfrica, y nos ayudarán a entender mejor la prevalencia de la enfermedad y el riesgo de contraerla.

### Asma

El asma es una enfermedad crónica común entre los niños de muchos países africanos. La prevalencia del asma infantil en África es mayor que el promedio global, y la incidencia de asma infantil está aumentando en África [45]. Al inicio de la pandemia, el asma se consideró un factor de riesgo para el COVID-19 grave. Sin embargo, datos recientes no indican que el asma aumente el riesgo de hospitalización ni de ingreso a la UCI por COVID-19 [32, 33]. En un estudio reciente de la Sociedad Respiratoria Europea, que incluyó a 945 niños con COVID-19 en 94 centros, 49 niños (5%) fueron asmáticos; la mayoría de los niños incluidos tuvieron síntomas leves, y 20% presentaron exacerbaciones clásicas del asma, potencialmente provocadas por el coronavirus. Aunque 9% de los niños asmáticos requirieron ingresar a la UCI, todos se recuperaron, con una estancia hospitalaria promedio de 6.9 ± 5.2 días [46]. De hecho, algunos centros reportaron un descenso en el número de ingresos por asma durante este periodo, probablemente debido a la combinación de una menor exposición a desencadenantes infecciosos durante el confinamiento, una mejor adherencia al tratamiento por temor a enfermar, y a la negativa de acudir a los servicios de salud [34, 35]. Nosotros experimentamos un descenso similar en los ingresos por asma en los inicios del confinamiento estricto, y no hemos visto el pico retardado que se esperaba debido a la falta de control. Sin embargo, a pesar de que el SARS-CoV-2 no parece causar comúnmente estornudos o una enfermedad grave [47], teóricamente es posible que los pacientes asmáticos sufran ataques desencadenados por el COVID-19; es esencial mantener un buen control del asma, como principio general, y como medida de precaución durante este tiempo.

### Fibrosis quística

Los pacientes con fibrosis quística (FQ) se consideraban en riesgo de contraer COVID-19 grave, especialmente aquellos con enfermedad pulmonar avanzada, con base en la experiencia anterior con FQ y la pandemia de influenza A (H1N1) en 2010 [48]. Sin embargo, un reporte preliminar de 8 países con 40 casos de COVID-19 en pacientes con FQ disipó la preocupación inicial [36]. La mayoría de los pacientes eran adultos (el más joven tenía 15 años), 38% tenían diabetes relacionada con la FQ, y 11 eran receptores de trasplante de pulmón. Trece pacientes (32%) requirieron oxigenoterapia, y uno (un receptor de trasplante pulmonar) requirió ventilación mecánica. No se reportaron muertes en esta serie. Los datos de la Sociedad Europea de FQ reportaron (hasta el 10 de septiembre de 2020) 144 casos de COVID-19 en pacientes con FQ, de los cuales solamente 36 eran <20 años, y todos tenían enfermedad leve o eran asintomáticos [37]. Hasta el momento, sólo se han documentado tres casos de COVID-19 en pacientes con FQ en Sudáfrica [49]. Se especula que una de las razones para la baja incidencia de COVID-19 en la población con FQ es su comportamiento preexistente, que incluye precauciones para evitar infecciones. Prevenir la infección es un principio importante en el cuidado de la FQ, que precedió a la pandemia. Sin embargo, la principal preocupación es el impacto perjudicial de la falta de cuidado continuo a la FQ en la salud de los pacientes. La FQ es una enfermedad compleja, que requiere atención multidisciplinaria y un monitoreo cercano. Usualmente, los pacientes acuden cada 1–3 meses para una revisión de rutina, de la función pulmonar y el análisis de esputo. Además, el tratamiento consiste en ejercicios diarios de fisioterapia torácica y la nebulización de medicamentos como los antibióticos. Desde que se implantaron las restricciones por el confinamiento debido al COVID-19, la atención médica regular se ha visto gravemente interrumpida, incluyendo la referencia rutinaria para pruebas de diagnóstico, y se suspendieron los programas de trasplante de pulmón. Más todavía, la mayoría de los pacientes y sus familiares evitan hasta donde es posible acudir a las instalaciones sanitarias. Ciertos procedimientos comunes para los pacientes con FQ, como las pruebas de función pulmonar, la recolección de muestras de esputo y la fisioterapia torácica con nebulizadores, se consideran de alto riesgo, debido a la aerosolización de gotas respiratorias. Aunque es razonable diferir las revisiones de rutina en algunos casos, la salud y el bienestar de los pacientes más vulnerables o enfermos pueden verse afectados. Esta preocupación se extiende a la investigación mundial sobre FQ, porque se han suspendido muchos ensayos clínicos activos [50]. Se han publicado lineamientos y recomendaciones para el cuidado de la FQ en Sudáfrica durante la pandemia de COVID-19, cubriendo todos los aspectos, desde cómo prevenir la infección por SARS-CoV-2, cómo reducir el riesgo de propagar el SARS-CoV-2, hasta cómo regresar a las escuelas o a los lugares de trabajo [51]; se señala la importancia prioritaria de reinstalar la atención continua a la FQ con medidas de control de la infección.

### Enfermedad pulmonar intersticial

Hay pocos datos sobre el impacto del COVID-19 en niños con enfermedad pulmonar intersticial (EPI), especialmente en LMICs. Una auditoría multicéntrica europea de pacientes mayores de 19 años con un diagnóstico de EPI preexistente y hospitalizados debido al COVID-19 mostró una mortalidad alta, de 39.3%, en pacientes con EPI subyacente, comparada con 15.4% en pacientes con edad, sexo y gravedad de COVID-19 similares, pero sin EPI preexistente [38]. Es muy probable que la gravedad de la enfermedad en estos pacientes se relacione con una combinación de EPI exacerbada por el COVID-19, una respuesta inflamatoria exagerada y anormalidades en la coagulación [38]. El uso de agentes inmunosupresores, que constituye el fundamento de la terapia en casos de EPI, no se ha asociado de manera consistente con la forma grave de COVID-19 [52]. De hecho, se ha encontrado que el uso de dexametasona mejora la supervivencia en el tratamiento de neumonía por COVID-19 y del síndrome de dificultad respiratoria aguda [53].

Por sí misma, la neumonía por COVID-19 puede ocasionar fibrosis intersticial. Se ha descrito el cuadro histopatológico de la neumonía por COVID-19 como el daño alveolar difuso del síndrome de dificultad respiratoria aguda, y se ha señalado que la fibrosis es una complicación terminal. La difusión gaseosa deficiente y la función pulmonar restrictiva son las anormalidades de la función pulmonar reportadas más comúnmente en estudios con cohortes de adultos luego de neumonía por COVID-19. El deterioro de la función pulmonar se relaciona con la gravedad de la enfermedad [39]. Ésta es una consideración importante en el seguimiento a largo plazo de los supervivientes de la forma grave del COVID-19 [54].

De manera similar a los reportes globales, ninguno de los pacientes con EPI ingresó en nuestra institución con COVID-19. Sin embargo, se requieren datos sobre el impacto del COVID-19 en niños con EPI preexistente. Dados los datos disponibles para los adultos, es razonable considerar que estos niños sufren un riesgo mayor y aconsejar medidas estrictas para controlar la infección.

### Traqueostomías

Tanto la inserción de una traqueostomía como su cuidado rutinario generan aerosoles, y por tanto presentan un riesgo elevado para la transmisión de COVID-19, especialmente para los trabajadores de la salud [55]. La mayor parte de la literatura sobre la inserción y el cuidado de una traqueostomía durante la pandemia de COVID-19 se refieren a adultos en entornos de ingresos altos. Se han publicado lineamientos sobre procedimientos seguros para la práctica y el cuidado de la traqueostomía en pacientes de COVID-19 [55, 56, 57]. Esto es importante, puesto que muchos pacientes con COVID-19 requieren ventilación prolongada y se benefician de la inserción de una traqueostomía (55, 56). Pueden aplicarse otras medidas para limitar la transmisión del virus durante el cuidado rutinario de la traqueostomía, incluyendo el uso de tubos para traqueostomía con manguito, circuitos de succión cerrados y filtros de calor y humedad [55, 57].

El sistema Breatheasy^©^ de RCWMCH es un programa holístico, multidisciplinario, que atiende a niños que requieren cuidado en casa para traqueostomía o ventilación de largo plazo [58, 59]. Hasta septiembre de 2020, el programa atendía a un total de 202 niños. Debido a la pandemia de COVID-19, las clínicas ambulatorias en este servicio migraron a una plataforma de telesalud, y sólo se mantuvieron las visitas hospitalarias esenciales. Únicamente dos pacientes dieron resultados positivos en una prueba de COVID-19 y tuvieron síntomas leves; en ambos casos, el contacto fue un familiar cercano. Esto sugiere que a pesar de que los niños con traqueostomía en general están en mayor riesgo por infecciones del tracto respiratorio inferior [60], no se observó COVID-19 grave en nuestra cohorte de pacientes.

Una fortaleza del programa Breatheasy^©^ es que favorece la organización de grupos de apoyo semanales para los cuidadores de niños admitidos en el hospital [59]. Esto crea una fuerte estructura de soporte psicosocial, particularmente para los cuidadores de niños que recibieron recientemente la traqueostomía, quienes interactúan con cuidadores más experimentados. Para facilitar el distanciamiento social se suspendió este grupo de apoyo, y esto trajo efectos negativos, evidenciados en un mayor estrés emocional y psicológico en los padres. La ansiedad en los cuidadores se agravó aún más por las regulaciones del hospital, que limitó la entrada y salida de cuidadores y visitantes del hospital. Estas limitaciones han tenido un impacto significativo en la dinámica familiar, especialmente en familias cuyos niños requirieron hospitalización prolongada para capacitarse en el cuidado de una traqueostomía.

## Tuberculosis (TB) y virus de la inmunodeficiencia humana (VIH)

El África subsahariana enfrenta la preexistencia de una epidemia doble de TB y VIH, además de la pandemia de COVID-19. Globalmente, 10 millones de personas desarrollaron TB en 2018; de éstas, 1.1 millones fueron niños, la mayoría de África subsahariana [61]. De los 1.8 millones de niños que viven con VIH, 90% están en África subsahariana [62]. Los niños infectados con VIH se encuentran en mayor riesgo de padecer enfermedades respiratorias agudas o crónicas, y pueden ser más susceptibles a la forma grave del COVID-19. Además, los infantes expuestos al VIH pero no infectados están en mayor riesgo de padecer infecciones del tracto respiratorio inferior, con peores resultados y en una época más temprana de su vida [63]; similarmente, pueden ser más susceptibles al COVID-19. La TB se asocia con un cuadro respiratorio crónico, lo que hace temer que también se asociará con un mayor riesgo de contraer COVID-19 y desarrollar la forma grave de la enfermedad. A la inversa, haber tenido TB podría, de hecho, aumentar la inmunidad innata, estimulando las respuestas inmunes al COVID-19, lo que constituye la base de la teoría de que la vacuna BCG podría brindar protección parcial contra la forma grave de la enfermedad [64]. La vacuna BCG aplicada al nacimiento, sin embargo, no parece conferir protección contra la infección por SARS-CoV-2 [65], aunque aún están en marcha estudios multicéntricos. No está claro si la infección latente por el bacilo de la tuberculosis y la coinfección con SARS-CoV-2 induce una tormenta inmunitaria, pero dos estudios de caso en China reportaron que la utilidad del uso de moduladores inmunes en pacientes adultos con TB latente, coinfectados con COVID-19 [66].

Los datos iniciales de África sugieren que la infección por VIH es un factor de riesgo para la gravedad de COVID-19 en adultos, aunque no es un hallazgo consistente en todo el mundo [40]. Una serie de casos de Italia no reportó al VIH como predictor para el COVID-19 ni para la muerte, especialmente en pacientes con supresión viral [67]. Un metaanálisis reciente mostró una mayor posibilidad de presentar la forma grave de COVID-19 en pacientes con inmunosupresión (776 pacientes; OR = 3.29, IC 95%: 0.89–12.21, *P* = 0.075), aunque las causas de inmunosupresión reportadas en ese estudio eran multifactoriales, no sólo VIH [68]. En Western Cape, Sudáfrica, el VIH y la presencia actual o previa de TB se asociaron de manera independiente con la muerte por COVID-19 en adultos, con un cociente de riesgos corregido (aHR) para VIH de 2.14, con un intervalo de confianza (IC) al 95% de 1.70–2.70, y un aHR para la TB actual y la previa de 2.70 (IC 95%, 1.81–4.04) y 1.51 (IC 95%, 1.18–1.93), respectivamente [40]. Sin embargo, la experiencia clínica hasta ahora no indica que el VIH o la TB sean factores de riesgo significativos para la muerte por COVID-19 en niños; no obstante, aún están analizándose los datos provinciales.

Con el confinamiento y la disminución de los servicios de salud que vemos en todo el mundo, se ha reducido el acceso de los pacientes a las instalaciones de salud para recoger los medicamentos de administración crónica para VIH/TB u otras enfermedades crónicas, tanto debido a las regulaciones del confinamiento como al temor de exponerse al COVID-19. Los servicios de tamizaje para otras condiciones comunes, como la TB, han sufrido competencia; p.ej., los cartuchos GeneXpert para TB se emplean para COVID-19 [69]; se da prioridad al análisis de COVID-19 en muestras de esputo, retrasando el diagnóstico de TB, y se emplea la vacuna BCG de manera inapropiada como respuesta a una teoría no-demostrada de que la vacuna BCG puede proteger contra el COVID-19 [64]. Se anticipa que ha ocurrido una reducción importante en el número de casos de TB detectados y verificados, y que los programas de tratamiento para estas enfermedades se verán interrumpidos, a menos que el programa de tamizaje y análisis de COVID-19 pueda llevarse a cabo simultáneamente con los programas para VIH/TB [70].

La presentación clínica del COVID-19, la TB o cualquier otra enfermedad respiratoria es no-específica. Los trabajadores sanitarios en áreas con alta incidencia deben insistir en el tamizaje estándar de TB y VIH en niños que presenten enfermedades del tracto respiratorio, además de las pruebas para COVID-19. Se requieren datos para entender mejor el riesgo no solamente de infección por VIH, sino también la exposición del VIH en el riesgo por COVID-19. Además, las implicaciones de la vacunación BCG, la TB previa y la coinfección actual con TB sobre la gravedad del COVID-19 en niños requiere mayor investigación.

## Sumario

El COVID-19 se asocia con una enfermedad más leve en niños en comparación con los adultos. A pesar de los temores iniciales sobre los niños con enfermedades pulmonares crónicas, la evidencia actual sugiere que una enfermedad respiratoria preexistente en niños no parece ser un factor de riesgo significativo para contraer COVID-19 grave. Esto tiene implicaciones importantes para la planeación del cuidado social y la educación. Aún se requieren estudios longitudinales y con conjuntos de datos más amplios para evaluar el riesgo en niños con inmunosupresión y con enfermedades pulmonares intersticiales, y deben tomarse precauciones con estos grupos. Se desconoce el efecto a largo plazo del COVID-19 en la salud pulmonar. Asimismo, no debe subestimarse el reconocimiento creciente del MIS-C en todo el mundo como causa importante de morbilidad y mortalidad relacionada con el COVID-19 en niños, pues se requiere identificarlo y tratarlo de manera temprana. Las medidas estrictas de salud pública que se aplican actualmente son esenciales para reducir el impacto de la pandemia en niños con enfermedades respiratorias y en sus familias. Más importante aún, los impactos indirectos de la respuesta a la pandemia en la salud respiratoria de los niños son notables, y aún hace falta comprenderlos y cuantificarlos. Garantizar que los niños tengan acceso a servicios preventivos y de cuidado completos durante este tiempo es prioritario.

## Financiamiento

DG recibió financiamiento de The Wellcome Trust y HZ de MRC South Africa. Número de subvención Gray Wellcome: 204755/Z/16/Z.

## Conflicto de interés

Los autores declaran que la investigación se realizó en ausencia de cualesquier relaciones comerciales o financieras que pudieran interpretarse como potencial conflicto de interés.

## Información sobre licencias

Diane M. Gray, Mary-Ann Davies, Leah Githinji, Michael Levin, Muntanga Mapani, Zandiswa Nowalaza, Norbertta Washaya, Aamir Yassin, Marco Zampoli, Heather J. Zar, Aneesa Vanker: COVID-19 and Pediatric Lung Disease: A South African Tertiary Center Experience. Front Pediatr. 2021 Jan 20;8:614076. ^©^ 2021 Los Autores (traducción; contribución de los autores abreviada), protegido por CC BY 4.0 (https://creativecommons.org/licenses/by/4.0/deed.es).

## Figures and Tables

**Table 1:** COVID-19 en niños con enfermedades respiratorias crónicas y comorbilidades respiratorias

**Enfermedad pulmonar crónica**	
Bronquiolitis obstructiva	Enfermedad pulmonar obstructiva crónica asociada con el COVID-19 grave en niños europeos [7, 30].
	
	Enfermedad pulmonar crónica, una comorbilidad común en pacientes ingresados por COVID-19 en Europa [7, 31].

Bronquiectasia	No se han publicado datos sobre bronquiectasia en COVID-19 pediátrico.

Asma	No aumenta el riesgo de hospitalización ni de ingreso a la UCI debido a COVID-19 [32, 33].
	
	Se ha reportado una reducción en el número de ingresos por asma en algunos centros [34, 35].
	
	El asma no-controlada es un riesgo para la mortalidad relacionada con COVID-19 en adultos [10]; se resalta la importancia de un buen control del asma.

Fibrosis quística	No está asociada con la forma grave del COVID-19 [36, 37].

Enfermedad pulmonar intersticial	Se desconoce el impacto en niños con EPI. La mayor mortalidad relacionada con COVID-19 en adultos con EPI sugiere tener precaución [38].
	
	La neumonía grave por COVID-19 es causa de daño alveolar difuso y fibrosis. Se ha reportado función pulmonar restrictiva y difusión gaseosa reducida en adultos después de neumonía por COVID-19 grave [39].

**Comorbilidades**	
Traqueostomía	No hay datos publicados sobre riesgo aumentado de infección o COVID-19 grave en niños con traqueostomía.
	
	Es probable que cualquier riesgo esté asociado con la condición subyacente.

Tuberculosis	La tuberculosis, actual o previa, se ha asociado con morbilidad por COVID-19 en adultos africanos [40]; actualmente no hay datos disponibles en niños.
	
	Hasta el momento, la experiencia clínica en niños no indica que la tuberculosis, sea infección o enfermedad, es un factor de riesgo significativo para la morbilidad y mortalidad por COVID-19. El análisis de los datos aún está en curso.

Infección por VIH	La infección por VIH es un factor de riesgo para la gravedad de COVID-19 en África (40); actualmente no hay datos disponibles en niños.
	
	Hasta la fecha, la experiencia clínica en niños no indica que el VIH ni la exposición a él sean factores de riesgo significativos para la morbilidad y mortalidad por COVID-19. El análisis de los datos aún está en curso.
